# Comparable Effects of High-Intensity Interval Training and Prolonged Continuous Exercise Training on Abdominal Visceral Fat Reduction in Obese Young Women

**DOI:** 10.1155/2017/5071740

**Published:** 2017-01-01

**Authors:** Haifeng Zhang, Tom K. Tong, Weifeng Qiu, Xu Zhang, Shi Zhou, Yang Liu, Yuxiu He

**Affiliations:** ^1^Physical Education College, Hebei Normal University, Shijiazhuang, China; ^2^Provincial Key Lab of Measurement and Evaluation in Human Movement and Bio-Information, Hebei, China; ^3^Department of Physical Education, Hong Kong Baptist University, Hong Kong; ^4^The Third Hospital of Shijiazhuang, Shijiazhuang, China; ^5^School of Health and Human Sciences, Southern Cross University, Lismore, NSW, Australia

## Abstract

This study compared the effect of prolonged moderate-intensity continuous training (MICT) on reducing abdominal visceral fat in obese young women with that of work-equivalent (300 kJ/training session) high-intensity interval training (HIIT). Forty-three participants received either HIIT (*n* = 15), MICT (*n* = 15), or no training (CON, *n* = 13) for 12 weeks. The abdominal visceral fat area (AVFA) and abdominal subcutaneous fat area (ASFA) of the participants were measured through computed tomography scans preintervention and postintervention. Total fat mass and the fat mass of the android, gynoid, and trunk regions were assessed through dual-energy X-ray absorptiometry. Following HIIT and MICT, comparable reductions in AVFA (−9.1, −9.2 cm^2^), ASFA (−35, −28.3 cm^2^), and combined AVFA and ASFA (−44.7, −37.5 cm^2^, *p* > 0.05) were observed. Similarly, reductions in fat percentage (−2.5%, −2.4%), total fat mass (−2.8, −2.8 kg), and fat mass of the android (−0.3, −0.3 kg), gynoid (−0.5, −0.7 kg), and trunk (−1.6, −1.2 kg, *p* > 0.05) regions did not differ between HIIT and MICT. No variable changed in CON. In conclusion, MICT consisting of prolonged sessions has no quantitative advantage, compared with that resulting from HIIT, in abdominal visceral fat reduction. HIIT appears to be the predominant strategy for controlling obesity because of its time efficiency.

## 1. Introduction

The prevalence of obesity and its related diseases has dramatically increased worldwide in recent decades. In contrast with the accumulation of peripheral subcutaneous fat, excessive fat accumulation in the abdominal viscera is strongly associated with obesity-related complications, including type 2 diabetes and cardiovascular disease [1]. The health crisis resulting from the adiposity that appears at these specific anatomical locations has been termed “metabolic obesity” [2]. The effective elimination of excessive abdominal fat in obese people through lifestyle modification and adjuvant appetite suppressants has been associated with reductions in fasting plasma glucose, triglycerides, and HOMA score [3]. Such beneficial effects on the parameters of metabolic syndrome induced by visceral fat reduction were more noticeable compared with those resulting from subcutaneous fat reduction [4].

There is robust evidence that high-volume, moderate-intensity continuous training (MICT) with exercise sessions ≥ 45 min can reduce abdominal visceral fat [3, 5–7] as well as improve body composition, cardiovascular fitness, and other health-related parameters including insulin sensitivity and lipid profile in both healthy and obese people [8, 9]. More recently, high-intensity interval training (HIIT), which consists of repeated high-intensity exercise bouts interspersed with passive/active recovery, was shown to induce similar metabolic adaptations associated with continuous training among healthy populations [10], as well as in patients with chronic diseases [11–13]. The interval training regime is furthermore assumed to be more advantageous than continuous endurance training in developing time-efficient lifestyle intervention strategies for controlling obesity [14]. However, the superiority of interval training in reducing abdominal fat, especially visceral fat, compared with continuous training has not been thoroughly studied. The contrasting findings regarding training-induced abdominal visceral fat reduction reported in previous studies might be associated with discrepancies in training protocols, including volume and intensity, methods of visceral fat measurement, obesity status, and gender [15–20]. Recently, we demonstrated the time-efficient advantage of HIIT in reducing the abdominal visceral fat of overweight young women, by comparing the program with a continuous training regime composed of 33-minute exercise sessions [20]. However, previous findings about the dominance of interval training did not specify whether comparison of the specific fat loss was conducted in obese individuals participating in continuous training with prolonged exercise sessions (≥45 min). A dose-response relationship of visceral fat adaptation to aerobic training was noted in obese individuals [21], whereas a similar dose-response effect from interval training was vague [22]. It is unknown whether continuous exercise training consisting of prolonged sessions would have a quantitative advantage in abdominal visceral fat reduction, compared with that resulting from a HIIT program, among obese young women.

This study examined the effects of 12-week HIIT and MICT programs on the reduction of whole-body, abdominal visceral, and abdominal subcutaneous fat, as assessed through dual X-ray absorptiometry (DEXA) and computed tomography (CT), in obese Chinese women. A randomized controlled trial was conducted to compare the specific HIIT adaptations with those resulting from a traditional MICT program with exercise sessions ≥ 45 min and with those of nonexercising counterparts. Participants in both HIIT and MICT groups each performed work equaling 300 kJ per exercise session. Notably, our study addresses a gap in scientific investigations that have examined exercise interventions with a high training volume and controlled amount of work in obese women and that quantify adiposity with direct imaging methods.

## 2. Methods

### 2.1. Participants

**Figure 1:**
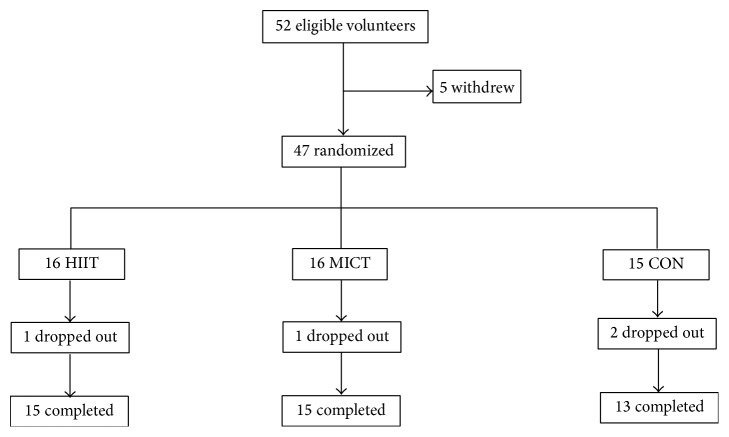
Distribution of study participants. HIIT, high-intensity interval training group; MICT, moderate-intensity continuous training group; CON, control group.

In total, 52 eligible female university students were recruited according to the following inclusion criteria: (1) age range of 18–22 years; (2) body mass index (BMI) ≥ 25 kg/m^2^; (3) body fat percentage ≥ 30 (as determined through DEXA); (4) body weight remained constant (±2 kg) during the past 3 months; (5) participation in a physical education class twice per week, but not in other regular physical activities or exercise training; and (6) no history of metabolic, hormonal, orthopedic, or cardiovascular diseases and no current use of prescribed medication. Five eligible students declined participation for personal reasons; the remaining 47 participants underwent initial assessment and randomization. During the intervention, four participants did not complete the program for reasons unrelated to the study (Figure 1).

Experiments in the present study were performed in accordance with the Helsinki Declaration. Following an explanation of the purpose and constraints of the study, the participants provided written informed consent. The Ethical Committee of Hebei Normal University for the Use of Human and Animal Subjects in Research provided ethical approval of the study.

### 2.2. Study Design

After preintervention assessments, participants with matching body fat percentages were randomly assigned to one of three groups. Group one received a MICT regime with prescribed work of 300 kJ in most training sessions; group two received an HIIT regime with work prescribed in each training session identical to that of MICT; group three was the control group (CON) that received no training. The changes in whole-body and regional abdominal fat, including in the abdominal visceral fat area (AVFA) and abdominal subcutaneous fat area (ASFA), resulting from these 12-week interventions were subsequently compared among the three groups. All participants were asked to maintain their daily activity and avoid altering their eating habits during the experimental period.

### 2.3. Exercise Training Protocol

In each training session, the MICT group participants performed continuous exercise on a cycle ergometer (Monark, 839E, Sweden) at an intensity of 60%  $\dot{V}O_{2max}$ until the targeted 300 kJ of work was achieved. By contrast, the HIIT group participants repeated 4-minute cycling exercise bouts at an intensity of 90%  $\dot{V}O_{2max}$, followed by a 3-minute passive recovery until the targeted 300 kJ of work was achieved. The pedal frequency was maintained at 60 rpm during each training session in both groups. Warm-up and cool-down exercises were standardized and identical in both groups. For the first 4 weeks, the participants in the two experimental groups exercised for 200 kJ (excluding warm-up and cool-down) for one session per day, 3 days per week. During the fifth through twelfth weeks, the training frequency was increased to 4 days per week, and the total work done in each session was increased to 300 kJ in both groups. All participants exercised with close supervision and exercise heart rate and perceived physical exertion (Borg scale 6–20) were monitored at every training session. Details of the exercise in a single session of HIIT and MICT are shown in Table 1. At the end of the fourth and eighth weeks, the $\dot{V}O_{2max}$ of all participants was determined to readjust the workload corresponding to a preset intensity. The training adherence of the participants was calculated as the percentage of the actual number of training sessions completed in compliance with the targeted intensity and duration, relative to the total number of training sessions prescribed.

### 2.4. Physical Activity and Dietary Assessments

A diary approach was used to estimate daily energy expenditures according to self-reported physical activity during a 3-week period prior to the intervention and during the twelfth week of the intervention. The total energy expenditure, expressed as metabolic equivalents (METs)·hr·wk^−1^, was estimated on the basis of the METs reported in the Compendium of Physical Activities [23] and the duration of recorded activities per week.

The diet (caloric intake) of each participant was recorded on a daily basis during the 3 weeks prior to the intervention and throughout the 12-week intervention period, according to the guidelines of the Sports Nutrition Centre of the National Research Institute of Sports Medicine (NRISM) in China. The dietary records and corresponding energy intake were analyzed each week by a dietician using the NRISM dietary and nutritional analysis system (version 3.1), designed for Chinese athletes and the general population. Dietary advice was provided to the participants by the dietician if the maintenance of the weekly caloric intake was violated.

### 2.5. Measurements

Total body mass; body fat percentage; fat mass of the whole-body, trunk, android, and gynoid regions; AVFA; ASFA; and aerobic fitness were measured 1 week before the start of the training program, as well as 3 days after the last training session. During the 2 days of measurements, the participants reported to the laboratory at 8:00 a.m. after a minimum 8-hour fast and refraining from strenuous exercise for 48 hours. The sequence of measurements, as follows, was identical preintervention and postintervention.

#### 2.5.1. Body Fat Measurement

Body weight and body fat percentage as well as the fat mass of the whole-body, trunk, android, and gynoid regions were measured through DEXA (Discovery Wi, Hologic Inc., Bedford, MA, USA). Regional demarcations were adjusted by a trained technologist according to guidelines described elsewhere [19]. Briefly, the trunk region included the area from the bottom of the neckline to the top of the pelvis, excluding the arms; the android region measured from the cut of the pelvic region to 20% of the distance between the pelvic cut and the bottom of the neckline, excluding the arms; and the gynoid region which was below the android region and had a height equal to two times that of the android region, with the pelvic cut as the upper demarcation. The intraclass correlation coefficients (ICCs) between two scans were >0.98, and the output from the DEXA scanner was the fat mass expressed in grams.

The cross-sectional AVFA and ASFA were assessed using a CT scanner (Somatom Definition Flash; Siemens, Forchheim, Germany), with a consistent acquisition protocol set at 120 kVp and 150 mA. During assessment, the participants laid in the supine position with their arms stretched above their heads; a 2 s, 5 mm scan was obtained from the umbilicus level (approximately the L4-L5 intervertebral space). The AVFA and ASFA were evaluated using the built-in volume calculation software of the CT scanner. For each scan, the number of voxels in the entire data set, with CT numbers between −190 and −30 HU, was plotted for adipose tissue. Examining the areas under the curve indicated the total adipose tissue volume. The ICCs for the AVFA and ASFA evaluations between two scans in selected participants were 0.94 and 0.95, respectively.

Identical body fat assessments were performed at the same time of the day preintervention and postintervention and in avoidance of the menses phases of the participants. The technicians responsible for the DEXA and CT measurements and analyses were also the same preintervention and postintervention and were unaware of the participants and intervention groups.

#### 2.5.2. Graded Exercise Test

The aerobic fitness of participants revealed by their $\dot{V}O_{2max}$ was determined using a graded cycling exercise protocol. The participants began at 50 W with a pedal frequency of 60 rpm; power output was increased by 30 W every 3 min until volitional exhaustion. Oxygen consumption during the exercise test was measured using a Cosmed breath-by-breath metabolic analyzer (Quark-PFT-ergo, Cosmed, Rome, Italy). $\dot{V}O_{2max}$ was calculated as the highest 30 s average value. The ICC of the $\dot{V}O_{2max}$ measurement in our laboratory was 0.92. Following the graded exercise test, a power output that elicited approximately 60% and 90%  $\dot{V}O_{2max}$ in the MICT and HIIT groups, respectively, was selected from the linear relationship of steady-state $\dot{V}O_{2}$ versus power output.

### 2.6. Statistical Analysis

The Shapiro-Wilk normality test revealed that all the data for body fat and aerobic fitness were normally distributed. A 2 × 3 two-way ANOVA with repeated measures was used to evaluate the main effects and interactions in the changes of body fat variables across all three groups (HIIT, MICT, and CON) between the preintervention and postintervention periods. Post hoc analyses using the Bonferroni test for identifying simple main effects were performed when a significant interaction was detected. A similar ANOVA was used to examine the differences in the habitual energy intake and energy expenditure across all groups between the preintervention and intervention periods. For where a significant interaction existed, planned comparisons (paired* t*-tests) with Bonferroni correction were performed to examine the difference between the preintervention and intervention periods in each group. All descriptive data were expressed as the mean ± SD. All tests for statistical significance were standardized at an alpha level of *p* ≤ 0.05.

## 3. Results

Of the 47 eligible participants, 43 (91%) completed the study interventions, comprising 15 of 16 participants (94%) from the HIIT and MICT groups and 13 of 15 participants (87%) from the CON group. The age and stature of the participants were not significantly different among the three groups (HIIT = 21.5 ± 1.7 y, 162.6 ± 4.6 cm; MICT = 20.9 ± 1.4 y, 162.8 ± 4.6 cm; CON = 20.8 ± 1.1 y, 160.0 ± 7.0 cm, *p* > 0.05). Among the participants who completed the study, compliance with the exercise intervention was 96%  ± 3% and 95%  ± 1% in the HIIT and MICT groups, respectively. No adverse events were reported during testing or training in either group.

### 3.1. Habitual Energy Intake and Energy Expenditure

The habitual energy intake and energy expenditure of the participants recorded during a 3-week period prior to the intervention and throughout the 12 weeks of the intervention are depicted in Table 2. No significant difference was noted within groups between the preintervention and intervention periods for habitual energy intake (*p* > 0.05); similar results were also found in the estimated energy expenditure for habitual physical activity, with exercise training being excluded during the intervention period.

### 3.2. Body Mass and Body Fat Percentage

The preintervention and postintervention body mass and body fat percentage of all three groups, as well as the repeated measures ANOVA results, are shown in Table 3. The baseline total body mass and body fat percentage exhibited no significant difference among the three groups (*p* > 0.05); however, after the 12-week intervention, a significant reduction in body mass and body fat percentage was observed in both intervention groups (*p* < 0.05), but not the CON group. The changes in body mass and body fat percentage did not differ significantly between the HIIT and MICT groups (*p* > 0.05).

### 3.3. Total and Regional Fat Mass

Table 3 presents the preintervention and postintervention measurements of whole-body fat mass and the fat mass of the android, gynoid, and trunk regions of all groups, as well as the repeated measures ANOVA results. The baseline fat mass variables did not differ significantly among the three groups (*p* > 0.05). However, subsequent to the 12-week intervention, significant reductions in all variables were noted among both the HIIT and MICT groups (*p* < 0.05); the CON group had no changes. The reductions in whole-body and regional fat mass did not vary between the two intervention groups (*p* > 0.05).

### 3.4. Abdominal Visceral and Subcutaneous Fat

The preintervention and postintervention AVFA, ASFA, and combined AVFA and ASFA of all groups, as well as the repeated measures ANOVA results, are also presented in Table 3. The baseline variables did not differ significantly among the three groups (*p* > 0.05).

**Figure 2:**
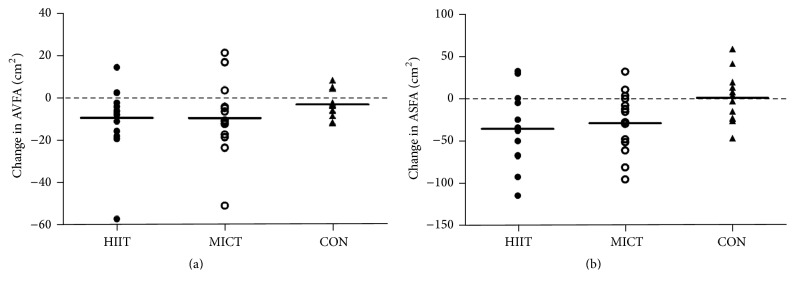
Changes in (a) abdominal visceral fat area (AVFA) and (b) abdominal subcutaneous fat area (ASFA) of participants postintervention in HIIT, MICT, and CON groups.

After the 12-week intervention, however, a reduction in the abdominal visceral fat revealed by an alternation in the AVFA from the corresponding baseline value (Figure 2(a)) was discovered in both intervention groups (*p* < 0.05); no change was noted in the CON group. The changes in variables between the intervention groups were not different (*p* > 0.05). A significant reduction also occurred in the ASFA, to a similar degree in both the HIIT and MICT groups postintervention (Figure 2(b)). Furthermore, a comparable reduction in overall abdominal fat (AVFA and ASFA) was observed in both the HIIT and MICT participants (*p* < 0.05). No change in the ASFA or the combined AVFA and ASFA was noted in the CON group.

### 3.5. Aerobic Fitness

The participants demonstrated increased aerobic fitness following the 12-week interventions. Postintervention $\dot{V}O_{2max}$ significantly increased in the HIIT (31.6 ± 2.2 versus 40.0 ± 4.5 mL·kg^−1^·min^−1^) and MICT (30.6 ± 3.5 versus 38.3 ± 4.4 mL·kg^−1^·min^−1^, *p* < 0.05) groups, while there was a small but significant decrease in the CON group (29.3 ± 3.5 versus 28.0 ± 3.2 mL·kg^−1^·min^−1^, *p* < 0.05). Between the two intervention groups, alternations in $\dot{V}O_{2max}$ were similar.

## 4. Discussion

Following the 12-week intervention, the participants in the work-equivalent HIIT and MICT exercise groups attained more than 10% reductions in the whole-body and regional (android, gynoid, and trunk) fat mass, as well as in the AVFA and ASFA (Table 3). The two exercise strategies also increased $\dot{V}O_{2max}$ measurements to a similar degree among the participants. The comparable fat loss and increased $\dot{V}O_{2max}$ results suggest that both HIIT and MICT regimes are effective measures for improving aerobic fitness and eliminating excess body fat in obese females. Notably, the exercise duration of more than 60 min per session in the MICT was almost double that of the interval exercise in the HIIT (Table 1); thus, despite a body fat elimination comparable with that from MICT regimes, the time-efficient characteristics of the HIIT possess a marked advantage in developing a habitual strategic exercise for combatting obesity.

Within the MICT group, reductions in whole-body and regional fat mass resulting from chronic prolonged continuous exercise corroborate previous findings of exercise-induced fat loss in healthy older adults and obese women [7, 19]. Similarly, the abdominal visceral fat loss observed in 12 out of 15 participants postintervention (Figure 2(a)) further supports other studies that have indicated that long-term, continuous aerobic exercise is associated with excess visceral fat elimination in obese people [3, 5]. Compared with our recent study, which examined participants involved in a shorter, 33 min per session MICT regime and indicated a less total O_2_ cost in a single session (50.7 ± 4.7 L versus 60.2 ± 3.4 L) [20], the present study revealed a much greater AVFA reduction (−4.8 cm^2^ versus −9.2 cm^2^). The improved visceral fat loss in the MICT regime of this study is in line with the previous notions of a systematic review of clinical trials for managing obesity [21]. Specifically, aerobic exercise-induced energy expenditure of 10 METs·hr·wk^−1^ (the current MICT regime expends approximately 17 METs·hr·wk^−1^) is the minimum level that would elicit significant visceral fat reduction in obese people without a metabolic-related disorder; increasing the aerobic exercise-induced energy expenditure would further augment the amount of visceral fat loss. The previously noted dose-response relationship (*r* = −0.75) between the aerobic exercise-induced energy expenditure and visceral fat reduction in metabolically healthy obese individuals suggested that the specific fat loss resulting from MICT is primarily dependent upon the training volume [21]. This further implies that the prolonged duration of exercise sessions is the foundation of MICT regimes for reducing abdominal visceral fat in obese people.

In contrast to MICT, the reduction in the AVFA following the prolonged HIIT in the present study did not appear in a greater extent (−9.1 cm^2^) when comparing to that (−11.8 cm^2^) resulting from the 12-week brief HIIT adopted in our recent study [20]. The brief protocol required the participants to perform only four high-intensity 4-minute runs in each session. The lack of advancement of the AVFA reduction resulting from the brief HIIT with a prolonged counterpart should not be attributed to the discrepancies in exercise mode and associated energy expenditure, because the total O_2_ expended for exercise recorded in a single session of the prolonged HIIT was approximately 10 L higher than that of the brief one (59.9 ± 2.1 L versus 49.8 ± 4.7 L). Although the difference in the baseline obesity status of participants may influence the outcomes of the two training regimes, the participants in the present study who possessed larger AVFAs (69.0 versus 64.9 cm^2^) did not take any advantage of their obesity in the resultant AVFA reduction. The lack of a dose-response effect of HIIT on visceral fat reduction is in agreement with recent findings that male sedentary adolescents performing extra HIIT sets did not achieve additional improvements in specific fat loss compared with that resulting from the lowest dose [22]. Apparently, HIIT-induced abdominal visceral fat reduction is not likely dependent upon the amount of energy expended in exercise sessions; this is further confirmed by our recent findings of a similar AVFA reduction of 9.7 cm^2^ (data not shown) in a group of young obese females after completing a 12-week HIIT at an identical intensity, where the work (400 kJ), exercise duration (46.3 ± 6.3 to 48.2 ± 6.4 min), and total O_2_ cost (81.2 ± 5.5 L) per session were greater than those of the present study.

The aforementioned differences in the adaptations of abdominal visceral fat in response to prolonged MICT and HIIT suggest that the underlying mechanisms for fat loss following the two training regimes may not be identical. An increase in exercise intensity is known to reduce the rate of fatty acid mobilization from adipose tissue into the blood, leading to a shift in the energy source from fat metabolism toward an increasing reliance on carbohydrates as a substrate [24]. The energy for repeating the high-intensity exercise sessions during the HIIT regime was thereby mainly generated by breaking down carbohydrates rather than lipolysis; nonetheless, the subsequent reductions in whole-body and regional fat mass following HIIT were equivalent to those that occurred after MICT (Table 3). This may be partly attributed to the greater elevation of the postexercise metabolic rate and associated fat expenditure in HIIT, because the magnitude and duration of the elevated postexercise O_2_ expended were reported to be greater following high-intensity exercise than after moderate exercise [25]. Compared with the brief HIIT adopted in our recent study [20], greater energy costs during and after exercise sessions in the prolonged HIIT regime of the current study are assumed. However, the greater training volume and possible associated total energy expenditure in the current HIIT program, which led to a greater reduction in whole-body fat (2.8 versus 1.9 kg), did not augment abdominal visceral fat loss, and our data were unable to elucidate the discrepancies in whole-body and visceral fat reduction. Nevertheless, HIIT regimes with similar exercise intensities but different training volumes resulted in a comparable reduction in the AVFA, suggesting that training volume may not be a relevant variable for modifying abdominal visceral fat storage.

Irving et al. [26] demonstrated the effect of endurance exercise training intensity on abdominal visceral fat reduction; however, it remains unclear whether the exercise intensity of HIIT is a critical variable that underlies the ability of specific training to reduce visceral fat. Lipolytic hormones including catecholamines and growth hormone have demonstrably increased with exercise intensity [27, 28]; specifically, catecholamines increase lipolysis in adipose tissues through *β*-adrenoceptors, which are more common in abdominal fat than in subcutaneous fat [29, 30]. Moreover, the elevation of plasma catecholamines induced by high-intensity intermittent exercise is higher compared with that which appears during steady-state exercise, potentially facilitating a reduction of abdominal fat [31]. Furthermore, the amount of fat expenditure in young adults during recovery from exercise was correlated with the intensity of the preceding exercise, and the increase in fat expenditure during recovery from higher exercise intensities was related to the release of growth hormone [28]; a clinical case of acromegaly clearly demonstrated that growth hormone promotes lipolysis in visceral fat [32]. In light of both current and previous findings, it is reasonable to postulate that the inclusion of high-intensity exercise sessions, rather than a high training volume, is the foundation of eliminating excess visceral fat in obese females with HIIT; however, how high the exercise intensity and how brief the training volume of HIIT for maximum efficiency in inducing significant modifications of the abdominal visceral fat of obese people are not clear. Previous studies [15, 16] have proposed administering 12–15-week HIIT programs that consist of alternating 8 s cycle sprints followed by 12 s low-intensity cycling for 60 cycles per session at three sessions per week to both healthy young women and overweight young men for fat loss. However, the effectiveness of these brief HIIT regimes in visceral fat reduction in the obese population has not been illustrated. Moreover, because training-induced changes in abdominal visceral fat are more manifest in men [18], future investigations on the minimum exercise intensity and training volume of HIIT required for a significant abdominal visceral fat reduction should be gender-specific.

In conclusion, both 12-week work-equivalent MICT and HIIT regimes successfully produced significant reductions in the fat mass of the whole-body and the android, gynoid, and trunk regions, as well as of the AVFAs and ASFAs in obese young females. The magnitude of the fat reduction was comparable between MICT and HIIT for each category, which indicates that neither training regime is superior at eliminating body fat. However, the higher training volume in both regimes resulted in contrary adaptations in visceral fat reduction in the homogeneous participants, compared with our prior findings [20]. Enhancement in visceral fat reduction with a higher training volume was found in MICT, but not in HIIT. This suggests that the inclusion of high training volumes is essential in MICT to eliminate excess visceral fat in obese females and indirectly verifies previous proposals that HIIT, rather than MICT, is the more practicable program for development into a habitual strategic exercise for combatting central obesity. For future investigations, the minimum quantity of training volume and its interaction with training intensity that could induce significant visceral fat reduction with HIIT are of interest.

## Figures and Tables

**Table 1:** The work, power, exercise time, heart rate (HR), and rating of perceived exertion (RPE) of training sessions in every four weeks during the 12-week intervention in HIIE and MICT groups.

	HIIT (*n* = 15)			MICT (*n* = 15)		
	Weeks 1–4	Weeks 4–8	Weeks 8–12	Weeks 1–4	Weeks 4–8	Weeks 8–12
Work (KJ)	200	300	300	200	300	300
Power (watt)	114.3 ± 15.4	133.8 ± 16.1	147.1 ± 16.4	66.5 ± 7.0	68.5 ± 10.4	80.4 ± 6.4
Exercise time (min)	29.4 ± 3.4	37.8 ± 4.6	34.4 ± 3.8	51.2 ± 5.8	74.4 ± 10.5	62.6 ± 5.0
HR (beats·min^−1^)	167.7 ± 12.8	169.7 ± 20.1	164.3 ± 6.1	140.2 ± 9.9	133.9 ± 8.7	136.8 ± 7.5
RPE	14.8 ± 1.1	15.6 ± 1.5	15.9 ± 1.8	11.8 ± 1.3	11.8 ± 1.3	11.9 ± 1.7

Values are means ± SD.

**Table 2:** The habitual energy intake and expenditure (excluding training) during the 3-week preintervention (Pre) and 12-week intervention (In) periods in HIIT, MICT, and CON groups.

	HIIT (*n* = 15)		MICT (*n* = 15)		CON (*n* = 13)	
	Pre	In	Pre	In	Pre	In
Habitual energy intake (Kcal·d^−1^)	1330 ± 408	1321 ± 403	1542 ± 584	1419 ± 455	1903 ± 315	1904 ± 315
Energy expenditure (METs·hr·wk^−1^)	50.4 ± 4.6	49.8 ± 4.2	56.6 ± 12.3	54.3 ± 11.9	79.4 ± 12.8	82.5 ± 11.5

Values are means ± SD.

The differences between Pre and In in each variable were not significant in all groups, *p* > 0.05.

**Table 3:** Pre- and postintervention levels and changes in body mass, % body fat, fat mass (FM) of whole-body, and regions of android, gynoid, and trunk, as well as abdominal visceral (AVFA) and subcutaneous (ASFA) fat areas in HIIT, MICT, and CON groups.

	HIIT (*n* = 15)		MICT (*n* = 15)		CON (*n* = 13)		2-way ANOVA *p* value (group, time, interaction)
	Pre	Post	Pre	Post	Pre	Post	
Body mass (kg)	67.3 ± 6.1	64.0 ± 6.0^*∗∗*^	68.5 ± 8.0	65.1 ± 7.7^*∗∗*^	67.5 ± 9.2	67.6 ± 9.2	0.80; 0.00; 0.00
	[−3.3 (−4.8, − 1.9)]^##^		[−3.4 (−5.0, − 1.8)]^##^		[0.1 (−1.2, 1.4)]		
% body fat (%)	38.1 ± 2.3	35.6 ± 2.0^*∗∗*^	38.0 ± 2.1	35.6 ± 2.3^*∗∗*^	40.9 ± 2.9	41.4 ± 3.0	0.00; 0.00; 0.00
	[−2.5 (−3.6, − 1.5)]^##^		[−2.4 (−3.3, − 1.4)]^##^		[0.5 (−0.6, 1.5)]		
Whole-body FM (kg)	25.7 ± 3.3	22.9 ± 3.1^*∗∗*^	26.1 ± 3.7	23.3 ± 4.0^*∗∗*^	27.8 ± 5.4	28.1 ± 5.1	0.04; 0.00; 0.00
	[−2.8 (−4.0, − 1.7)]^##^		[−2.8 (−3.8, − 1.7)]^##^		[0.3 (−0.8, 1.4)]		
Android FM (kg)	2.0 ± 0.4	1.7 ± 0.4^*∗∗*^	1.9 ± 0.3	1.6 ± 0.3^*∗∗*^	2.2 ± 0.4	2.2 ± 0.4	0.00; 0.00; 0.00
	[−0.3 (−0.4, − 0.2)]^##^		[−0.3 (−0.4, − 0.2)]^##^		[0.0 (−0.2, 0.2)]		
Gynoid FM (kg)	4.6 ± 0.5	4.1 ± 0.6^*∗∗*^	4.8 ± 0.9	4.1 ± 0.8^*∗∗*^	4.8 ± 1.1	4.9 ± 1.0	0.19; 0.00; 0.00
	[−0.5 (−0.7, − 0.4)]^##^		[−0.7 (−0.9, − 0.4)]^##^		[0.1 (−0.1, 0.2)]		
Trunk FM (kg)	11.7 ± 2.1	10.1 ± 2.1^*∗∗*^	11.4 ± 2.1	10.2 ± 2.0^*∗∗*^	13.1 ± 2.2	13.0 ± 1.9	0.01; 0.00; 0.01
	[−1.6 (−2.2, − 0.9)]^##^		[−1.2 (−1.8, − 0.7)]^#^		[−0.1 (−1.0, 0.9)]		
AVFA (cm^2^)	69.0 ± 24.7	59.9 ± 19.1^*∗*^	69.4 ± 26.6	60.2 ± 23.5^*∗*^	69.7 ± 20.3	66.9 ± 22.6	0.87; 0.00; 0.40
	[−9.1 (−17.8, − 0.4)]		[−9.2 (−18.5, 0.11)]		[−2.8 (−6.5, 0.9)]		
ASFA (cm^2^)	248.4 ± 61.2	213.4 ± 51.0^*∗∗*^	219.9 ± 47.6	191.6 ± 35.5^*∗∗*^	285.5 ± 70.3	287.1 ± 63.3	0.00; 0.00; 0.02
	[−35.0 (−57.4, − 12.7)]^#^		[−28.3 (−47.8, − 8.8)]^#^		[1.6 (−15.7, 18.9)]		
AVFA + ASFA (cm^2^)	317.4 ± 72.2	273.3 ± 56.5^*∗∗*^	289.3 ± 63.0	251.8 ± 48.9^*∗∗*^	355.3 ± 79.6	354.1 ± 75.1	0.00; 0.00; 0.02
	[−44.1 (−70.9, − 17.4)]^##^		[−37.5 (−64.2, − 10.7)]^#^		[−1.2 (−19.4, 17.0)]		

Values are means ±SD [mean change (95% confidence interval)] .

^*∗*^ *p* < 0.05; ^*∗∗*^*p* < 0.01, significantly different from corresponding Pre value.

^#^ *p* < 0.05; ^##^*p* < 0.01, significantly different from corresponding CON value.
